# Expression of TweakR in breast cancer and preclinical activity of enavatuzumab, a humanized anti-TweakR mAb

**DOI:** 10.1007/s00432-012-1332-x

**Published:** 2012-10-17

**Authors:** Debra T. Chao, Mian Su, Sonia Tanlimco, Mien Sho, Donghee Choi, Mel Fox, Shiming Ye, Eric D. Hsi, Lisa Durkin, Johnny Yin, Yongke Zhang, Han Kim, Gary C. Starling, Patricia A. Culp

**Affiliations:** 1 Discovery, GPRD, Abbott Biotherapeutics, 1500 Seaport Blvd, Redwood City, CA 94063 USA; 2 Department of Clinical Pathology, Cleveland Clinic, Cleveland, OH USA

**Keywords:** TweakR, Antibody, Breast cancer, Fn14

## Abstract

**Background:**

The receptor for the cytokine TWEAK (TweakR) is a cell surface member of the tumor necrosis factor receptor superfamily with diverse biological roles. TNFRSF family members are appealing therapeutic targets in oncology due to their aberrant expression and function in tumor cells. The goal of the current study was to examine the potential of TweakR as a therapeutic target in breast cancer.

**Methods:**

Expression of TweakR in primary breast cancer tissues and metastases was characterized using immunohistochemistry. To determine the functional relevance of TweakR, breast cancer cell lines were treated in vitro and in vivo with enavatuzumab, a humanized mAb against TweakR.

**Results:**

Overexpression of TweakR was observed in infiltrating tumors compared to normal adjacent breast tissues, and strong staining of TweakR was observed in all subtypes of invasive ductal breast cancer. In addition, a positive correlation of TweakR and HER2 expression and co-localization were observed, irrespective of ER status. TweakR expression was also observed in bone metastasis samples from primary breast cancer but rarely in benign tumors. Enavatuzumab inhibited the in vitro growth of TweakR-expressing breast cancer cell lines, and this activity was augmented by cross-linking the mAb. In addition, enavatuzumab significantly inhibited the in vivo growth of multiple breast cancer xenograft models including a model of metastasis.

**Conclusions:**

TweakR is highly expressed in all subtypes of invasive ductal breast cancer, and enavatuzumab administration exhibited a dose-dependent inhibition of primary tumor growth and lung metastasis and enhanced the antitumor activity of several chemotherapy agents currently used to treat breast cancer. These data provide the rationale to evaluate enavatuzumab as a potential therapy for the treatment of breast cancer.

**Electronic supplementary material:**

The online version of this article (doi:10.1007/s00432-012-1332-x) contains supplementary material, which is available to authorized users.

## Introduction

Breast cancer is the most common cancer in women worldwide. An estimate of more than 1.6 million new cases of breast cancer occurred among women in 2010 (Forouzanfar et al. 2011). Prognosis and selection of therapies are often influenced by clinical and pathological features including expression of specific tumor markers such as ER/PR and HER2. In addition to standard chemotherapies, endocrine therapies (e.g., estrogen receptor antagonists and aromatase inhibitors) are used to treat ER-positive tumors, while trastuzumab (anti-HER2 mAb) is a standard of care for treating HER2-positive cases. Even though significant progress has been made in the treatment of breast cancer, there remains a need for new treatments, especially for patients with more aggressive tumors and in advanced stages. In fact, metastasis of primary breast tumor cells to distant organs is responsible for the majority of breast cancer deaths (Hurvitz 2011).

TWEAK receptor (TweakR, Fn14, TNFRSF12A) is a cell surface protein and member of the tumor necrosis factor receptor superfamily and is activated by its ligand, TWEAK (Wiley et al. 2001; Winkles 2008). TweakR was first described as an FGF-inducible gene which played a role in fibroblast adhesion and migration (Meighan-Mantha et al. 1999). Subsequently, the induction of TweakR expression by other growth factors and/or upon tissue injury was observed in multiple cell types, including hepatocytes, endothelial cells, adipocytes, and cardiomyocytes. These observations and additional studies have suggested that the TWEAK/TweakR interaction may play a role in tissue repair and inflammation (Campbell et al. 2004; Chacon et al. 2006; Chorianopoulos et al. 2010; Feng et al. 2000; Jakubowski et al. 2005). The initial identification of TWEAK as a weak inducer of apoptosis, along with a role in the regulation of migration and angiogenesis, demonstrates the pleiotropic nature of the TWEAK/TweakR system (Harada et al. 2002; Jakubowski et al. 2002; Lynch et al. 1999; Nakayama et al. 2002; Schneider et al. 1999; Wiley et al. 2001).

Previously, we have reported the overexpression of TweakR in many types of solid tumors and demonstrated the therapeutic potential of a mAb targeting TweakR (enavatuzumab, formerly PDL192) (Culp et al. 2010). Here, we expand the analysis to the role of TweakR in breast cancer. We show that TweakR is overexpressed on different subtypes of primary patient samples and demonstrate a correlation of TweakR and HER2 expression. We also provide a functional rationale for the use of enavatuzumab in the treatment of breast cancer with in vitro and in vivo studies on breast cancer cell lines.

## Materials and methods

### Tissues and immunohistochemistry staining

Immunohistochemistry analysis for TweakR was performed on primary breast cancer samples as previously described (Culp et al. 2010). Matched normal adjacent and cancer tissues were collected from surgery specimen of breast cancer patients by Zoion Diagnostic (Shrewsbury, MA) with appropriate IRB approval at participating clinical sites. TweakR staining was scored based on percentage of epithelial cells stained in the sample (0, <10 %; 1+, 11–25 %; 2+, 26–50 %; 3+, 51–75 %; 4+, >75 %). Two hundred and thirty-seven samples were also stained for HER2 using anti-HER2 mouse mAb (clone CB11, BioCare Medical, Concord, CA) and for estrogen receptor (ER) using anti-ER mouse mAb (clone 1D5, BioCare Medical). Samples were considered HER2 positive with IHC score ≥2+; ER was considered positive if the sample had >10 % of epithelial cells stained positive. All stainings were scored by a board-certified oncology pathologist.

Co-staining of TweakR and HER2 was performed in 40 invasive ductal breast cancer samples on a tissue microarray with the two-step procedure. First, the samples were stained for TweakR expression with an internal mouse mAb (374.2) and detected by MACH4-HRP system; subsequently, the samples were stained with a rabbit mAb (EP1045Y, Biocare Medical) for HER2 expression and detected with MACH4-AP system.

### Cell lines and reagents

All tumor cell lines were purchased from American Type Culture Collection (Manassas, VA) except the BT549 and MX1 cell lines, which were obtained from DTP/DCTD Tumor Repository at the National Cancer Institute (Frederick, MD), and the MB231 variant cell line, which was derived from the MDA-MB-231 cell line for its increased metastatic potential in vivo. All purchased cell lines were maintained in the growth medium recommended by the vendor, and the MB231 variant cell line was maintained in IMDM supplemented with 10 % fetal bovine serum.

The anti-TweakR monoclonal antibody 374.2 was generated by immunizing Balb/c mice with a synthetic peptide comprising amino acids 37–49 (SRGSSWSADLDKC) of the human TweakR coding region. Hybridomas were screened for TweakR specificity by Western blot and immunohistochemistry (IHC) on samples with known levels of TweakR expression. Enavatuzumab, a humanized anti-TweakR antibody, was previously described (Culp et al.). The human IgG1 control antibody, MSL109, is a fully human antibody to cytomegalovirus (Drobyski et al. 1991). The cross-linking secondary antibody was purchased from Jackson Immunoresearch (Cat# 109-006-008, West Grove, PA). Trastuzumab (Herceptin^®^) was purchased from Genentech (South San Francisco, CA).

TweakR was measured on cell lines by flow cytometry using enavatuzumab, followed by a fluorescein isothiocyanate-conjugated anti-human secondary antibody (Life Technologies, Carlsbad, CA). Expression was reported as the fold increase in mean fluorescence intensity versus control antibody-stained cells. Cell lines were confirmed to be of the HER2, luminal, or basal subtype by measuring ErbB3, EGFR, E-cadherin, EpCAM, HER2, and CD44 expression by flow cytometry (see Supplemental Methods, Supplemental Table 2). Expression of additional luminal or basal-specific genes (Finn et al. 2009; Neve et al. 2006) was assessed in a subset of cell lines by microarray analysis (Supplemental Table 3).

### Growth inhibition assay

Proliferation assays were performed by incubating 300–2,000 cells in 96-well plates with enavatuzumab in the presence or the absence of a cross-linking secondary antibody at a 2:1 (primary: secondary) ratio. Cell density per well was determined based on growth property for each cell line. For samples treated with immobilized antibody, ELISA plates (Immulon^®^ 4HBX, Thermo Scientific, Rochester, NY) were coated with antibody at the indicated concentrations, incubated for 24 h at 4 °C, and washed three times with PBS prior to plating cells. When untreated cells were ~80 % confluent, five to 10 days after plating, cell viability was assayed using resazurin (25 μM, #R7017, Sigma, St. Louis, MO) for 2 h at 37 °C. Relative viability was calculated by dividing the viability of anti-TweakR antibody-treated samples with that of control antibody-treated cells. In most cases, assay results represent the readings from triplicate wells. Each assay was performed at least twice, and representative data are shown.

For synergy analysis with trastuzumab, 500 SKBR3 cells were seeded in 96-well plates, and antibodies were added 24 h later. Cells were incubated with enavatuzumab (10–0.006 μg/mL) with half-molar ratio of cross-linking antibody, trastuzumab alone (1–0.0006 μg/mL), or titrations of one antibody in the presence of constant concentrations of the other. The presence of cross-linking antibody had no effect on the activity of trastuzumab. Ten days later, cell viability was assessed with resazurin (Sigma).

Synergy analysis was performed by calculating the combination index (CI) at multiple effect sizes using the Chou–Talalay method (Chou 2010): CI = (Ce_comb_/Ce_alone_) + (Ct_comb_/Ct_alone_), where Ce = the concentration of enavatuzumab and Ct = concentration of trastuzumab, to achieve a particular effect size. CI < 0.9 = synergy, 1.1 ≥ CI ≥ 0.9 = additivity, and CI > 1.1 = antagonism. Synergy was observed at the majority of antibody concentrations at effect sizes from 30 to 60 % inhibition, with smaller CI obtained at larger effect sizes (Supplemental Figure 1). CI could not be calculated at effect sizes greater than 60 % inhibition, as trastuzumab monotherapy did not result in more than 60 % inhibition.

### Xenograft tumor studies

Tumor cells were inoculated into the mammary fat pad of 6-week-old severe combined immunodeficient (SCID) mice (IcrTac:ICR-Prkdc^<scid>^, Taconic, Germantown, NY) at 1 × 10^7^ cells per mouse. For the MCF-7 model, animals were also implanted with 17β-estradiol pellets (0.36 mg, 60 day release, Innovative Research of America, Sarasota, FL). Animals were randomized into groups when the mean tumor volume reached 100–120 mm^3^. Antibodies were administered intraperitoneally three times per week for 3 weeks. Gemcitabine (Gemzar^®^, Eli Lilly, IN) and vinorelbine (Navelbine^®^, Sicor, Irvine, CA) were dosed intraperitoneally twice a week for three weeks. Tumor volumes (LxWxH/2) were measured on dosing days; the group means + SEM are displayed. Groups were removed from the study when at least one tumor in the group reached the allowable limit (1,500 mm^3^). The statistical significance of the differences between groups was determined by *T* test using SAS statistical software. Mean tumor volumes between groups were considered significantly different if *p* ≤ 0.05. All animal work was carried out under NIH guidelines “Guide for the Care and Use of Laboratory Animals” using IACUC approved protocols.

Lung metastases were quantified in the MB231 variant study on day 37. Mouse lungs (5 animals per dose group) were fixed in formalin and stained for human cytokeratin 7/8 (Cam5.2 antibody) at Mosaic Laboratories (Lake Forest, CA). The tissues were scanned using ScanScope software (Aperio, Vista, CA), and cytokeratin-positive cells were quantified using ImageScope software (Aperio). Both the numbers of micrometastases (individual cells and clusters of less than 4 cells) as well as the numbers of metastases with more than 4 cells per cluster were quantified.

## Results

### TweakR expression is elevated in invasive ductal breast tissues

**Fig. 1:**
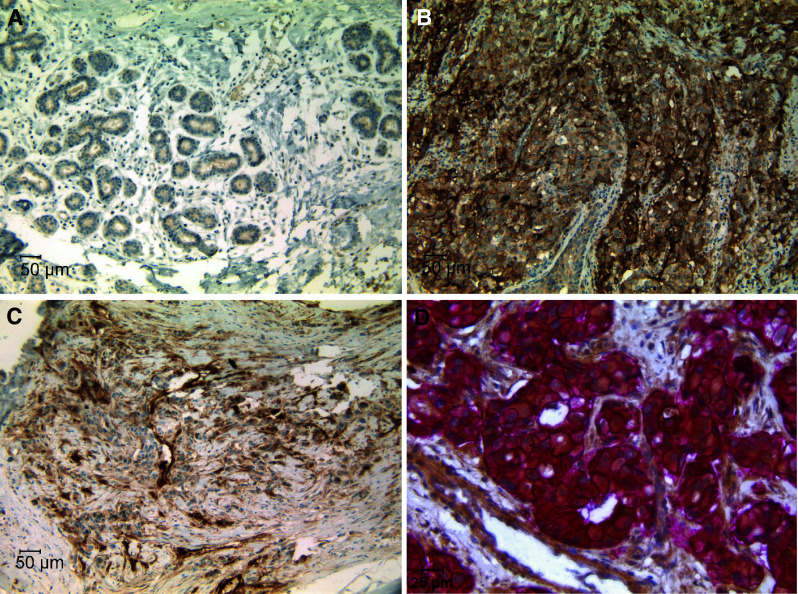
TweakR is expressed in invasive breast cancer samples and is co-expressed with HER2. Immunohistochemistry staining for TweakR expression was performed in normal adjacent tissue (**a**) and infiltrating tumor tissue (**b**) from the same case of breast cancer (200× magnification). **c** TweakR expression in bone metastases from breast cancer (200× magnification). **d** Double staining of TweakR (*brown*) and HER2 (*Red*) in a ductal breast cancer sample (400× magnification). Representative images are shown here

TweakR expression was evaluated in 522 paraffin samples of primary breast tissues and metastases by immunohistochemistry (IHC). Over thirty percent (119/378) of invasive ductal breast cancer had strong staining of TweakR, but only 1 out of 46 cases of lobular breast cancers was positive (Supplemental Table S1). TweakR expression was evaluated on eleven matched pairs of breast cancer and normal adjacent tissue. TweakR was detected in nine of eleven cases, and in each case, TweakR expression was elevated in the infiltrating tumor compared to normal tissue (Fig. 1a, b). In some tumor samples, TweakR staining was observed on stroma cells and the vasculature in addition to the tumor epithelial cells (data not shown).

Development of breast cancer progresses through stages of hyperplasia, benign lesions, and DCIS before the tumors become invasive. Lymph nodes and bones are two common organs for breast cancer metastasis. No TweakR staining was observed on a panel of hyperplasia or benign breast tissues, and only one of 18 DCIS cases was positive. In contrast, TweakR expression was detected in 5 out of 10 bone metastases (Fig. 1c) and 3 out of 30 lymph node samples from patients with metastatic breast cancer (Supplemental Table 1). These findings are consistent with a previous report of high TweakR expression correlating with metastatic breast cancer (Willis et al. 2008).

### TweakR expression correlates with HER2 expression

Breast cancers are often categorized into different subtypes according to their HER2 and ER status. A subset of the invasive breast cancer samples (237 cases) that had been stained for TweakR expression was also stained for HER2 and ER to determine whether their expression correlated with TweakR expression. TweakR expression was considered positive if the staining was observed on more than 25 % of epithelial cells **(**IHC score ≥ 2+). Using the chi-square test of independence, TweakR and HER2 expression were found to be significantly associated, with a *p* value of 1.129E−10 (Table 1). Approximately 60 % of HER2-positive cases also expressed TweakR, while fewer than 25 % of HER2-negative cases stained positive for TweakR. On the contrary, no such correlation was observed between TweakR and ER expression. These observations are consistent with previously published data (Willis et al., 2008). Co-immunostaining of TweakR and HER2 was performed on a subset of TweakR+/HER2+ breast cancer samples to determine whether TweakR and HER2 were expressed in the same cells within a tumor. In these samples, membranous HER2 staining clearly coincided with cytoplasmic and membranous TweakR staining in the majority of tumor cells (Fig. 1d).

**Table 1:** Positive correlation of TweakR expression with HER2 overexpression

Type	N	TWEAKR IHC score					≥2	(%) positive
		0	1	2	3	4		
ER +/HER2+	63	14	12	11	16	10	37/63	59
ER +/HER2−	51	34	5	5	4	3	12/51	24
ER-/HER2+	57	9	10	7	17	14	38/57	67
ER-/HER2−	66	37	16	6	3	4	13/66	20
Total	237	94	43	29	40	31	100/237	42

Using the chi-square test, TweakR and HER2 expression were found to be significantly associated (*p* value = 1.129E−10)

### In vitro growth inhibition by enavatuzumab is enhanced upon cross-linking in all subtypes of breast cancer cell lines

**Fig. 2:**
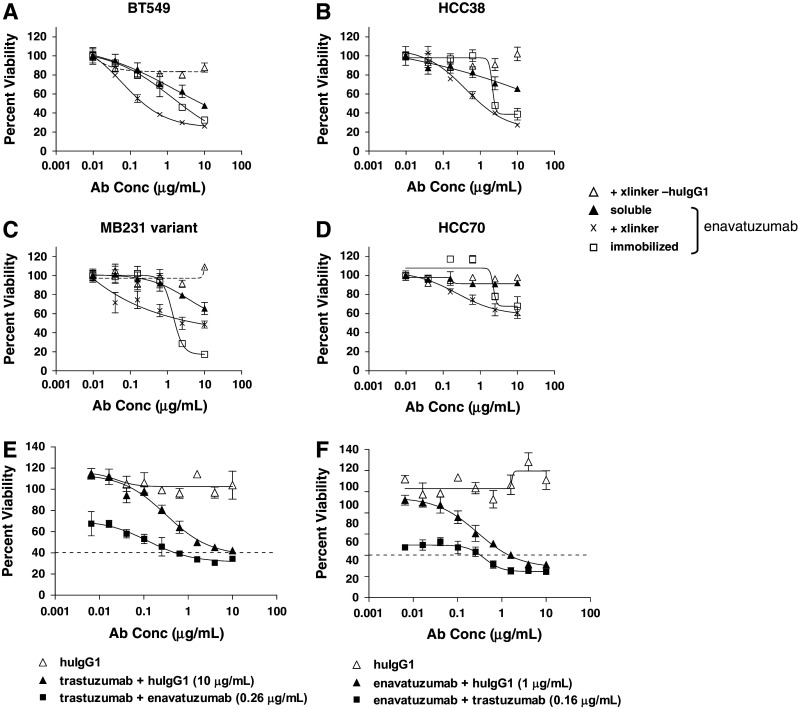
Growth inhibition of breast cancer cell lines by enavatuzumab and synergy of inhibition when combined with trastuzumab. **a**–**d** BT549 (**a**), HCC38 (**b**), MB231 variant (**c**), and HCC70 (**d**) breast cancer cells were incubated with soluble enavatuzumab or human IgG1 control antibody in the presence (x, Δ) or absence (*filled triangle*, not shown) of cross-linking antibody, or immobilized enavatuzumab or control antibody (*filled square*, not shown) for 5–10 days. Relative viability was calculated by dividing the viability of treated cells by that of untreated cells. Representative data are shown here (point, mean of triplicate wells; *bars*, SEM). All experiments were repeated at least twice (E+F). SKBR3 cells were incubated with titrations of trastuzumab (**e**) or enavatuzumab with cross-linking secondary antibody (**f**) in the presence or absence of a single concentration of the other antibody (0.16 μg/mL trastuzumab and 0.26 μg/mL enavatuzumab are shown). At the effect size of 60 % inhibition (*dashed line*), the combination index (CI) was 0.27 for the enavatuzumab + trastuzumab combination and 0.28 for the trastuzumab + enavatuzumab combination, suggesting significant synergy between the two antibodies. Synergy was observed at the majority of antibody concentrations tested at effect sizes from 30 to 60 % inhibition (Supplemental Figure S1)

Enavatuzumab is a humanized anti-TweakR antibody that exhibits potent antitumor activity in vitro and in vivo on cell lines derived from a variety of tumor types (Culp et al. 2010). To characterize further the functional activity of enavatuzumab in breast cancer, a panel of TweakR-expressing breast cancer cell lines was evaluated for sensitivity to enavatuzumab in proliferation assays in vitro. This panel included cancer cell lines reflecting all subtypes of breast cancer, as previously defined by their molecular profile (Finn et al. 2009; Hu et al. 2009; Hurvitz and Finn 2009; Neve et al. 2006). Expression of HER2 and luminal- or basal-specific markers was confirmed on the panel by flow cytometry and/or microarray analysis (Supplemental Table S2 and S3). In general, subtype classification of the cell lines was in agreement with that reported by others. The breast cancer cell lines were treated with enavatuzumab in a soluble form, cross-linked in solution with a secondary antibody, or immobilized enavatuzumab. Soluble enavatuzumab significantly and reproducibly inhibited the growth of 5 of 27 cell lines by >20 %, while cross-linked or immobilized enavatuzumab had more potent effects in a larger subset of cell lines, 13 of 27 lines and 18 of 27 cell lines exhibited >20 % growth inhibition, respectively (Table 2; Fig. 2a). Cross-linking increased both the number of cell lines sensitive to enavatuzumab and the potency of the antibody, as evidenced by the relative decrease in EC_50_ values of cross-linked versus soluble antibody treatment. In contrast, while immobilization significantly increased the maximal inhibition by enavatuzumab over cross-linked antibody, the EC_50_ increased for most cell lines when the antibody was immobilized versus cross-linked antibody (Table 2). The higher EC_50_ likely reflects a dependence on physical proximity between adjacent immobilized antibody molecules to enable cross-linking of cell surface TweakR molecules. Sensitivity to enavatuzumab was observed in all subtypes of breast cancer expressing antigen yet did not appear to correlate with TweakR expression levels, as measured by flow cytometry (Table 2).

**Table 2:** Enavatuzumab inhibits breast cancer cell growth in vitro

	Subtype	TweakR expression	Inhibition of proliferation by enavatuzumab					
			Soluble		+xlinker		Immobilized	
			10 μg/mL	EC_50_ (μg/mL)	10 μg/mL	EC_50_ (μg/mL)	10 μg/mL	EC_50_ (μg/mL)
BT20	B	7.5	–	−	−	−	+	1.21
BT474	H	2.3	−	−	−	−	±	1.85
BT483	L	2.5	−	−	+	2.30	+++	1.48
BT549	B	4.7	+	1.32	++	0.20	++	0.72
Cama1	L	3.8	−	−	+	1.57	++	2.16
DU4475	B	1.5	−	−	−	−	−	−
HCC1143	B	3.4	−	−	+	0.18	+	1.31
HCC1428	L	1.4	−	−	±	1.85	+	1.77
HCC1500	B	2.7	−	−	±	1.75	+++	1.60
HCC1569	H	2.8	−	−	±	0.32	−	−
HCC1937	B	5.3	±	4.46	+	0.42	+	2.38
HCC38	B	6.6	+	0.31	++	0.31	++	1.53
HCC70	B	2.4	−	−	+	0.23	±	2.30
Hs578T	B	4.8	−	−	−	−	−	−
MB231 variant	B	8.3	+	2.98	+	0.15	++	1.33
MCF7	L	5.8	−	−	±	0.41	+	1.27
MDA-MB157	B	5.9	−	−	±	0.06	+	1.20
MDA-MB175 VII	L	1.4	−	−	−	−	−	−
MDA-MB231	B	10.3	+	1.99	+	0.35	+	1.02
MDA-MB361	H	4.7	−	−	−	−	−	−
MDA-MB453	H	2.6	−	−	+	1.04	+	2.59
MDA-MB468	B	8.8	+	0.31	+	0.52	+++	1.10
MX1	B	4.7	−	−	+	0.71	++	1.08
SKBR3	H	2.3	±	1.93	+	0.50	++	1.36
T47D	L	1.8	−	−	−	−	−	−
ZR-75-1	L	1.9	−	−	−	−	+	1.48
ZR-75-30	H	1.6	−	−	−	−	−	−

*B* basal, *L* luminal, *H* HER2+

TweakR expression: fold increase of TweakR versus control

Inhibition score: − <10 %, ±10–20 %, +20–50 %, ++50–75 %, +++ >75 %, *nt* not tested

### Enavatuzumab synergizes with trastuzumab in vitro

Given the observation that enavatuzumab inhibited the growth of breast cancer cell lines in vitro and that TweakR expression correlated with HER2 overexpression in primary breast cancers, enavatuzumab was assessed for its potential to synergize with trastuzumab, the standard of care for HER2-positive breast cancer. SKBR3 cells (HER2+) were exposed to titrations of enavatuzumab alone, trastuzumab alone, or both drugs simultaneously in ratios sufficient to assess cooperative interaction (Chou 2010). Synergy in tumor cell growth inhibition was observed with the combination of enavatuzumab and trastuzumab, as the Combination Index was found to be less than 1.0 at multiple effect sizes (Fig. 2b, Supplemental Figure S1).

### Enavatuzumab does not stimulate invasion or migration in vitro

TweakR has been shown to play a role in tumor cell migration and invasion in vitro, as expression of TweakR appears to correlate with the invasive potential of breast cancer cell lines (Willis et al. 2008). In addition, TWEAK, the natural ligand of TweakR, has been shown to stimulate the adhesion, migration, and invasion of cancer cell lines (Dai et al. 2009; Tran et al. 2003). Enavatuzumab can activate signaling pathways downstream of TweakR, but has moderate agonist activity compared to TWEAK (Culp et al. 2010). Given the ability of TWEAK to stimulate migration and invasion of tumor cells, enavatuzumab was assessed for its ability to stimulate migration or invasion of breast cell lines in vitro. Cell lines with detectable but basal levels of invasiveness were assessed in an invasion assay, while cell lines with low innate invasive potential were assessed in a migration assay. TWEAK stimulated migration/invasion of 4 of 7 cell lines tested, while enavatuzumab did not stimulate invasion of any cell lines and enhanced the migration of just one cell line tested, MB453 (Supplemental Table S4, Supplemental Figure S2). Of the seven evaluated, soluble enavatuzumab had inhibited the proliferation of three cell lines. For one such line, SKBR3, TWEAK also enhanced invasion. Notably, enavatuzumab did not significantly stimulate invasion by this cell line (Supplemental Figure S2).

### Therapeutic effect of enavatuzumab in xenograft models of breast cancer

**Fig. 3:**
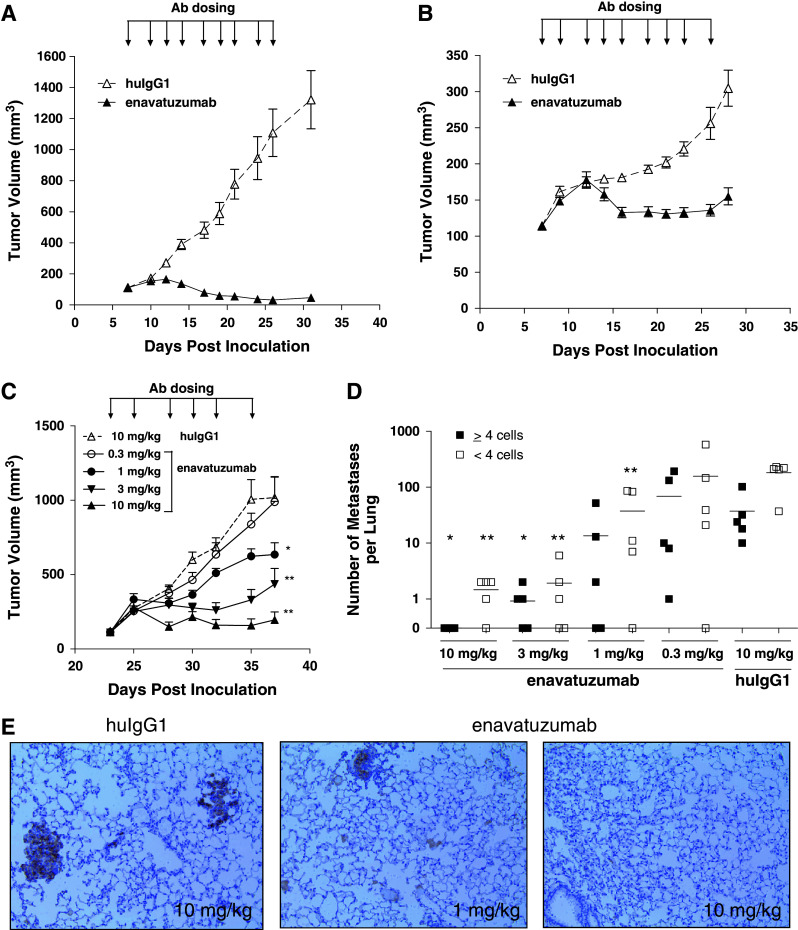
Enavatuzumab inhibited the growth of primary tumors and metastases in xenograft models of breast cancer. Established MCF7 (**a**) and HCC70 (**b**) tumors were treated with enavatuzumab or a human IgG1 control antibody at 10 mg/kg three times per week, with 10 animals in each dosing group. Tumor volumes were measured on each dosing day; points, mean; *bars*, SEM. Differences in tumor volumes between the enavatuzumab and control treated groups were significant (*p* < 0.001) in both models. **c**–**e** Established MB231 variant tumors were treated with enavatuzumab or a human IgG1 control antibody thrice per week, with 7 mice in each dosing group. **c** Tumor volumes were measured on each dosing day; points, mean; bars, SEM. **d** Metastases were quantified in lungs harvested on day 37; both micrometastases (<4 cells) and metastatic clusters >4 cells were enumerated. Enavatuzumab treatment significantly inhibited the growth of primary tumors and micrometastases at 10, 3, and 1 mg/kg and inhibited the development of larger metastases at 10 and 3 mg/kg (*p < 0.05; **p < 0.001). **e** Immunohistochemical staining of human cytokeratin-positive metastases in mouse lungs

In a previous study, enavatuzumab was found to exhibit potent antitumor activity in mouse xenograft models derived from several tumor types. However, as no breast cancer models were tested in those studies, enavatuzumab was next evaluated for its potential therapeutic effect in breast cancer models in vivo. Enavatuzumab exhibited potent antitumor activity in multiple xenograft models, including MCF-7, an ER-positive breast cancer cell line (Fig. 3a), and HCC70, a triple-negative (ER^−^/PR^−^/HER2^−^) line (Fig. 3b). In the MCF-7 model, enavatuzumab induced tumor regression in all animals, with complete eradication observed for 5/10 tumors. In the HCC70 model, enavatuzumab largely stabilized tumor growth. In a third model, the triple-negative MB231 variant model, enavatuzumab was assessed for its effect on both primary tumor growth and on the development of metastases. In this model, enavatuzumab treatment suppressed primary tumor growth in the mammary fat pad, with a clear dose–response relationship (Fig. 3c). In addition, enavatuzumab significantly inhibited the development of lung metastases in this model (Fig. 3d, e). The growth of micromets (<4 cells) and macromets (≥4 cells) were similarly inhibited by enavatuzumab.

**Fig. 4:**
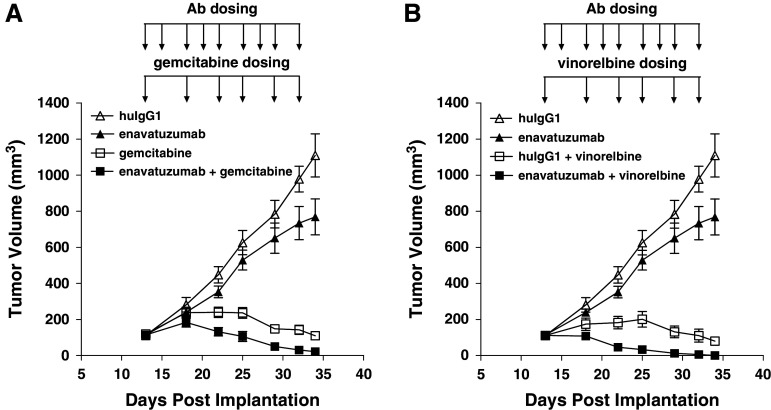
Enavatuzumab enhanced the antitumor activity of gemcitabine and navelbine in a triple-negative model of breast cancer. Established MB231 variant xenograft tumors were treated with enavatuzumab at 1 mg/kg thrice weekly, alone or in combination with gemcitabine at 30 mg/kg (**a**) or vinorelbine at 5 mg/kg (**b**) twice weekly, with 7 mice in each dosing group. Tumor volumes were measured on gemcitabine and vinorelbine dosing days; points, mean; *bars*, SEM. Differences in tumor volumes were significant (*p* < 0.05) between the gemcitabine and enavatuzumab/gemcitabine treatment groups and the vinorelbine and enavatuzumab/vinorelbine treatment groups on days 22–34. The data shown in **a** and **b** were derived from a single study. A second study yielded similar results

In the MB231 variant model, enavatuzumab was also tested in combination with gemcitabine and vinorelbine, agents that are commonly used to treat patients with triple-negative breast cancer. A suboptimal dose of enavatuzumab (1 mg/kg) was used in this study and alone had no significant impact on tumor growth (Fig. 4). However, enavatuzumab at this dose level enhanced the antitumor effects of both gemcitabine and vinorelbine, with complete eradication observed in 3 of 10 mice in the enavatuzumab/gemcitabine combination and in 7 of 7 tumors with the enavatuzumab/vinorelbine combination (Fig. 4).

## Discussion

Our study of the TweakR IHC analysis covered a wide range of breast tissue samples showing minimal TweakR expression in normal, hyperplasia, benign, DCIS, and invasive lobular breast cancer tissues while demonstrating increased TweakR expression in invasive ductal breast cancer, LN, and bone metastases from breast cancer patients. TweakR expression was found to be overexpressed on all subtypes of invasive ductal breast cancer, and TweakR positively correlated with HER2 overexpression, consistent with a previous report (Willis et al. 2008). Our novel finding that these proteins are co-expressed in breast tumor epithelial cells and the observation that enavatuzumab and trastuzumab synergize in inhibiting the growth of HER2-positive breast cancer cells in vitro suggests a functional interaction between these two proteins. Synergy in vitro was observed in multiple cell lines, but only in trastuzumab sensitive, HER2 overexpressing lines (SKBR3, ZR-75-30, and MDA-MB-453); however, synergy was unable to be confirmed in vivo, as none of these cell lines formed tumors reproducibly to enable xenograft studies. HER2 overexpressing cell lines appeared to be innately less sensitive to enavatuzumab than other subtypes, as 2 of 6 HER2 overexpressing lines were sensitive to soluble, cross-linked, or immobilized antibody. However, the finding of synergy with trastuzumab suggests that this subset of patients may benefit from the addition of enavatuzumab to trastuzumab-based therapy.

Some of the cell lines with the greatest sensitivity to enavatuzumab included those of the basal subtype, also categorized as triple-negative tumors, as they lack expression of estrogen receptor, progesterone receptor, and HER2. The standard therapy for patients with basal-type tumors is cytotoxic chemotherapy, as no targeted therapies currently exist for this subtype, and this patient population generally has poor prognosis (Cleator et al. 2007). Basal-type tumors can be further subdivided into those that retain the epithelial phenotype (as defined by expression of GATA3, TOB1, and cytokeratins) and those that have undergone epithelial-to-mesenchymal transition and express genes typical of the mesenchymal phenotype, including vimentin, CD44, and Axl (Neve et al. 2006). These tumors tend to be highly invasive and appear to have stem cell-like qualities. The finding that several cell lines with a mesenchymal phenotype (BT549, MDA-MB231, and MB231 variant, Supplemental Table 3) are highly sensitive to enavatuzumab in vitro suggests that this antibody may provide a promising therapeutic for this population of patients for whom current therapies offer limited benefit.

The basal-type MB231 variant cells were also quite sensitive to enavatuzumab treatment in vivo. These tumors are highly invasive and metastasize readily to the lung. In this model, enavatuzumab not only inhibited primary tumor growth, but also significantly inhibited the growth of tumors at metastatic sites. In this model, metastases are generally not detectable until the primary tumor is ~500 mm^3^ (data not shown), and the antibody treatments that resulted in the most dramatic decrease in the growth of metastases (3 and 10 mg/kg) also prevented the primary tumors from reaching 500 mm^3^. Thus, enavatuzumab treatment may inhibit the growth of lung metastases through several possible mechanisms including inhibition of extravasation, induction of cell death during migration, or inhibition of tumor seeding and/or growth in the lung.

Importantly, metastatic growth was not enhanced by enavatuzumab. TweakR overexpression has been shown to enhance the invasive properties of breast cancer cells in vitro, and stimulating TweakR signaling with TWEAK promotes tumor cell migration (Dai et al. 2009; Tran et al. 2003; Willis et al. 2008). The inability of enavatuzumab to stimulate invasion in vitro or metastasis in vivo may be due to its reduced agonist activity, compared to TWEAK, and/or may reflect the inability of in vitro assays to replicate fully complex in vivo processes.

Previous studies have shown that enavatuzumab is a partial agonist, directly inhibiting the growth of tumor cells in vitro (Culp et al. 2010). This inhibitory activity has been shown to be not through induction of apoptosis, the mechanism first ascribed to TWEAK, but via cell cycle arrest through activation of the NFkB pathway and upregulation of p21 (Purcell et al. 2012) In the current studies, growth inhibition by enavatuzumab in vitro was enhanced in most cell lines when the antibody was cross-linked, either in solution or by immobilization. Such enhancement of activity by antibody cross-linking has previously been shown for TweakR and other members of the TNF receptor superfamily (Alzona et al. 1994; Law et al. 2005; Mori et al. 2004; Nakayama et al. 2003). Antibody cross-linking likely enhances the formation of receptor multimers, which are thought to drive downstream signaling for this family of receptors (Motoki et al. 2005). Cross-linking of antibodies in vitro is likely to be physiologically relevant, as Fc receptors on immune and other cells provide endogenous cross-linking capability for antibodies in vivo (Wilson et al. 2011). Indeed, enavatuzumab treatment in multiple xenograft models has been found to enhance tumor infiltration of immune cells, which would enable Fc–Fc receptor (Fc–FcR) interactions (Ye et al. 2012). For several antibodies, Fc–FcR interactions have been shown to have therapeutic consequences, as patients bearing high-affinity alleles of FcγRIIIA exhibit increased responses to trastuzumab and rituximab compared to patients with low-affinity alleles (Cartron et al. 2002; Varchetta et al. 2007; Weng and Levy 2003).

While one functional consequence of enhanced Fc–FcR interactions may be increased antibody-dependent cellular cytotoxicity (ADCC), for some cell surface proteins, receptor clustering and enhanced downstream signaling may also result (de Haij et al. 2010). In the present study, enavatuzumab exerted only modest growth inhibition on ER + MCF7 cells in vitro, and only when the antibody was immobilized. However, in vivo, enavatuzumab treatment resulted in regression of MCF7 tumors (Fig. 3a). The apparent discrepancy between responses in vitro and in vivo is likely a result of in vivo Fc–FcR interactions, as a version of enavatuzumab containing a mutation preventing Fc–FcR interactions did not inhibit the growth of MCF7 tumors (data not shown). Fc–FcR-dependent activity of enavatuzumab in the MCF7 model may be a result of ADCC, enhanced downstream signaling, or a combination of both.

TweakR expression did not correlate directly in response to enavatuzumab in vitro. However, TweakR is upregulated by serum and other growth factors (Meighan-Mantha et al. 1999), and the vast majority of human breast cancer cell lines tested expressed significant levels of TweakR under standard growth conditions in vitro which does not necessarily reflect the tumor growth environment in vivo. Indeed, TweakR expression was observed in a smaller subset of primary breast cancer samples than in cultured cells in vitro. Thus, it is not clear whether the level of TweakR expression might correlate with sensitivity to enavatuzumab in vivo although a minimal level of TweakR expression is necessary for efficacy.

The observation that TweakR is expressed in multiple subtypes of breast cancer and that enavatuzumab exhibits potent in vitro and in vivo activity, both as monotherapy and in combination with other agents, provides the rationale for further evaluation of enavatuzumab for the treatment of breast cancer.

## Electronic supplementary material

Below is the link to the electronic supplementary material.

Supplementary material 1 (DOCX 14 kb)

Supplementary material 2 (DOCX 37 kb)

Figure S1. Enavatuzumab and Trastuzumab Synergy Analysis. Synergy analysis was performed by calculating the combination index (CI) at multiple effect sizes using the Chou-Talalay method: CI = (Cecomb/Cealone) + (Ctcomb/Ctalone), where Ce = the concentration of enavatuzumab and Ct = concentration of trastuzumab, to achieve a particular effect size. CI < 0.9 = synergy, 1.1 ≥ CI ≥ 0.9 = additivity, and CI > 1.1 = antagonism. CI could not be calculated at effect sizes greater than 60 % inhibition, as trastuzumab monotherapy did not result in more than 60 % inhibition(DOCX 84 kb)

Figure S2. TweakR Agonists Have Differential Effects on Invasion (A) and Migration (B). SKBR3 (A) or MCF7(B) tumor cells were loaded into the top chambers of wells either coated (A, invasion assay) or not coated (B, migration assay) with Matrigel in the presence of either enavatuzumab or huIgG1 (10 μg/m) or TWEAK (100 ng/m). Assay medium containing 5 % FCS was added to the bottom chambers. After overnight incubation, cells were stained with Calcein AM and quantified. Each experiment was performed at least two times, with one representative experiment shown. *p < 0.05 (DOCX 78 kb)
