# Supplementary material for: Expression of TweakR in breast cancer and preclinical activity of enavatuzumab, a humanized anti-TweakR mAb
Source: J Cancer Res Clin Oncol. 2012 Oct 17;139(2):315–25. doi: 10.1007/s00432-012-1332-x (PMC3549414; doi:10.1007/s00432-012-1332-x)
Supplement: Supplementary file 1 — Supplementary material 1 (DOCX 14 kb) [file 432_2012_1332_MOESM1_ESM.docx]

### Supplemental Methods

### Expression analysis

Cell lines were assessed for expression of luminal/basal markers by flow cytometry using antibodies to EpCAM (Catalog #347197, Becton Dickenson, Mountain View, CA), E-cadherin (Catalog #3199S, Cell Signaling, Danvers, MA), ErbB3 (Catalog #FAB3481P, R&D Systems, Minneapolis, MN), EGFR (Erbitux®, Imclone, Bridgewater, NJ), HER2 (Herceptin®, Genentech, South San Francisco, CA), and CD44 (Catalog #555479, BD). Expression is reported as the fold increase in mean fluorescence intensity relative to cells stained with control antibodies. Expression of additional luminal and basal markers was assessed in eleven cell lines by microarray analysis, as previously described ([Henshall et al., 2003](#_ENREF_16)). An average intensity (AI) unit below 50 is considered background.

### Migration and Invasion assay

Measurement of tumor cell migration and invasion was performed using the BD HTS FluoroBlok 96-well transwell system with an 8-um pore size (BD Biosciences Discovery Labware, Bedford, MA). Briefly, tumor cells (1x10^4^) were loaded into the top chambers of wells either coated (invasion assay) or not coated (migration assay) with Matrigel in the presence of either enavatuzumab or MSL109 (10 μg/mL) or TWEAK (100 ng/mL). Assay medium containing 5% FCS was added to the bottom chambers. After overnight incubation, cells migrating or invading through Matrigel were stained with Calcein AM, and cell images were acquired and quantified using Discovery-1 (Molecular Devices, Philadelphia, PA). Each experiment was performed at least two times, with one representative experiment shown.
