# Supplementary material for: Expression of TweakR in breast cancer and preclinical activity of enavatuzumab, a humanized anti-TweakR mAb
Source: J Cancer Res Clin Oncol. 2012 Oct 17;139(2):315–25. doi: 10.1007/s00432-012-1332-x (PMC3549414; doi:10.1007/s00432-012-1332-x)
Supplement: Supplementary file 2 — Supplementary material 2 (DOCX 37 kb) [file 432_2012_1332_MOESM2_ESM.docx]

**Supplemental Tables**

**Table S1. TweakR Expression is Associated with Breast Cancer Progression**

|  |  | **TweakR IHC Score** | | | | |  |  |
| --- | --- | --- | --- | --- | --- | --- | --- | --- |
| **Type** | **N** | **0** | **1** | **2** | **3** | **4** | **≥2** | **% pos.** |
| **Normal** | **16** | **16** | **0** | **0** | **0** | **0** | **0/16** | **0** |
| **Hyperplasia** | **12** | **12** | **0** | **0** | **0** | **0** | **0/12** | **0** |
| **Benign** | **12** | **12** | **0** | **0** | **0** | **0** | **0/12** | **0** |
| **DCIS** | **18** | **16** | **1** | **1** | **0** | **0** | **1/18** | **6** |
| **Invasive Ductal** | **378** | **183** | **76** | **41** | **46** | **32** | **119/378** | **32** |
| **Invasive Lobular** | **46** | **45** | **0** | **0** | **1** | **0** | **1/46** | **2** |
| **LN Mets** | **30** | **22** | **5** | **2** | **1** | **0** | **3/30** | **10** |
| **Bone Mets** | **10** | **2** | **3** | **0** | **5** | **0** | **5/10** | **50** |

DCIS, Ductal Carcinoma In Situ; LN, lymph Nodes; Mets, Metastases

**Table S2. Expression of HER2, Luminal, and Basal Markers by Flow Cytometry Analysis**

**Table S3: Expression of HER2, Luminal, and Basal Markers by Microarray Analysis**

**Table S4: TweakR Agonists Have Differential Effects on Mobilization of Breast Cancer Cells**

| **Cell Line** | **Assay** | **Activity** | |
| --- | --- | --- | --- |
|  |  | **TWEAK** | **enavatuzumab** |
| **SKBR3** | Invasion | + | - |
| **MB231 variant** | Invasion | - | - |
| **BT549** | Invasion | - | - |
| **BT20** | Invasion | - | - |
| **MB453** | Migration | + | + |
| **BT474** | Migration | + | - |
| **MCF7** | Migration | + | - |

‘+’ Stimulatory activity; ‘-’ no significant activity observed
