# Supplementary material for: Expression of TweakR in breast cancer and preclinical activity of enavatuzumab, a humanized anti-TweakR mAb
Source: J Cancer Res Clin Oncol. 2012 Oct 17;139(2):315–25. doi: 10.1007/s00432-012-1332-x (PMC3549414; doi:10.1007/s00432-012-1332-x)

Supplemental Figure S2: TweakR Agonists Have Differential Effects on Invasion and Migration

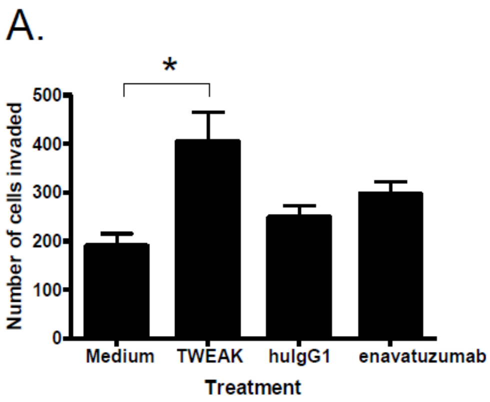

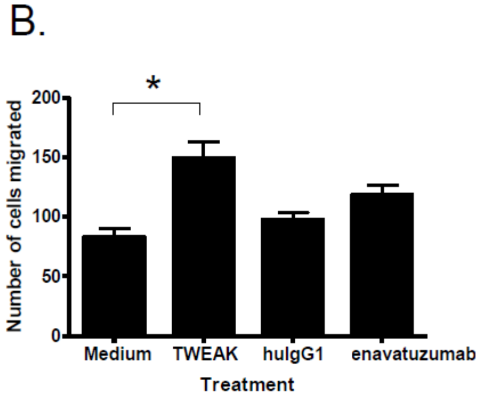

Supplement: Supplementary file 4 [file 432_2012_1332_MOESM4_ESM.docx]
